# The third-generation anti-CD30 CAR T-cells specifically homing to the tumor and mediating powerful antitumor activity

**DOI:** 10.1038/s41598-022-14523-0

**Published:** 2022-06-21

**Authors:** Shangkun Zhang, Chaojiang Gu, Lifang Huang, Han Wu, Jiangzhou Shi, Zijian Zhang, Yong Zhou, Jingjiao Zhou, Yang Gao, Jiaxing Liu, Yingqi Leng, Xiyu Liu, Qinxing Zhang, Liang Huang, Xiqin Tong, Ken H. Young, Jiapeng Li, Haichuan Zhu, Tongcun Zhang

**Affiliations:** 1 Institute of Biology and Medicine, College of Life and Health Sciences, Wuhan University of Science and Technology, Hubei, 430081 China; 2 Department of Hematology, Tongji Hospital, Tongji Medical College, Hua Zhong University of Science and Technology, Wuhan, 430030 China; 3 Department of Hematology, Zhongnan Hospital of Wuhan University, Wuhan, 430071 China; 4 Division of Hematopathology, Department of Pathology, Duke University Medical Center, Durham, NC 27710 USA; 5 Key Lab of Industrial Fermentation Microbiology, Ministry of Education, Tianjin Key Lab of Industrial Microbiology, College of Biotechnology, Tianjin University of Science and Technology, Tianjin, 300457 China

**Keywords:** Hodgkin lymphoma, Immunology, Adaptive immunity

## Abstract

CAR T-cell therapy is well tolerated and effective in patients with Hodgkin lymphoma (HL) and anaplastic large cell lymphoma (ALCL). However, even second- generation anti-CD30 CAR T-cells with CD28 (28z) costimulatory domains failed to achieve the desired rate of complete responses. In the present study, we developed second-generation (CD28z) and third-generation (CD28BBz) CAR T-cells targeting CD30 and investigated their efficacy in vitro and in vivo. Both of CD28z and CD28BBz anti-CD30 CAR T cells were similar regarding amplification, T cell subsets distribution, T cell activity, effector/memory and exhaustion. However, we found that the 28BBz anti-CD30 CAR T-cells persist long-term, specifically homing to the tumor and mediating powerful antitumor activity in tumor xenograft models. Subsequently, we also demonstrated that the third generation anti-CD30 CAR T-cells have miner side effects or potential risks of tumorigenesis. Thus, anti-CD30 CAR T-cells represent a safe and effective treatment for Hodgkin lymphoma.

## Introduction

Hodgkin lymphoma (HL) is a B cell malignancy with an excellent prognosis for most patients^1^. However, about 10–30% of patients will suffer from relapse and refractory disease^2,3^. Some of the patients who are resistant to initial HL therapy typically receive salvage chemotherapy followed by high-dose chemotherapy and autologous stem cell transplantation (ASCT)^4,5^. Although the outcome of these treatments has been improving over time, nearly half of such patients are not cured and have limited options for treatments maintaining long-term survival^3,6^. Currently, treatment with a novel targeted drug brentuximab vedotin (BV) [Adcetris], which is an antibody conjugate drug targeting CD30, has resulted in objective anti-tumor responses^7–9^. Results from a Phase II study of single agent brentuximab vedotin were an 75% ORR and 34% CR rate, respectively^10^. However, antibody therapy has limited bio-distribution and its benefits may be short-lived, so it is imperative to explore other approaches employing single or combination treatments with brentuximab vedotin to improve its anti-tumor activity for r/r CD30+ lymphoma patients. 

Adoptive immunotherapy is an approach strategy for the treatment of cancer that showed promise in recent trials^11,12^. The chimeric antigen receptor (CAR) cell-based adoptive immunotherapy strategy, in which T cells are engineered to express synthetic receptors, has thus far been employed predominantly for the treatment of hematological malignancies^13^. For example, CAR T-cells targeting CD19 demonstrated remarkable efficacy for the treatment of B cell malignancies^14,15^. For HL, CD30 is an excellent candidate as a target of CAR T-cell therapy due to its abundant and specific expression on the HL tumor cells but limited expression on normal tissues.

The first CAR T-cells used for targeting CD30+ HL cell lines in vitro can be traced back to the 1990s^16^. These CAR molecular constructs possessed the extracellular single-chain variable fragment (scFv) but lacked an intracellular costimulatory signaling domain, which limited their antitumor activity. In recent years, two groups published results of a phase I study of autologous CD30 CAR T-cell infusion. Although the scFv, intracellular costimulatory signals, delivery system, preconditioning regiments and doses were different in these two studies, both of them showed remarkable efficacy in CD30+ patients with chemotherapy-resistant HL and ALCL^17,18^. In their clinical trials, Wang and colleagues evaluated the response following anti-CD30 CAR T-cell infusion in 18 patients with r/r HL and reported an overall response rate (ORR) of 39%^17^, while Ramos et al. reported nine patients who with a 33% ORR and the efficacy may due to the higher disease burden which seven out of nine patients resistance to several cycling brentuximab treatment manifested^18^. Moreover, both studies documented a high degree of tolerability and safety in r/r HL and ALCL after CAR T-cell infusion^18^.Recently, Ramos et al. followed two parallel phase I/II studies involving patients with r/r HL and administered CD30 CAR T-cells. The ORR in 32 patients who received fludarabine-based lymphodepletion was 72% including a CR rate of 59%. However, 1-year progression-free survival for all evaluable patients was only 36% indicating that most patients had relapsed 1 years after CAR T-cell infusion^19^. The clinical results indicated that different binding affinities of scFv and intracellular constructs which are the essential elements for CAR-T antitumor activity need to be explored in order to improve outcomes in r/r HL and ALCL.

A wide variety of clinical trials showed that third-generation CARs exhibit improved effector functions and in vivo persistence as compared to the second-generation CARs^20–22^. In the previous study, we incorporated CD28 and 4-1BB (CD137) in addition to CD3ζ and selected an scFv to create third-generation anti-CD30 CAR T-cells. We found that the third-generation CAR mediated robust activity in r/r HL and ALK- ALCL, yielding an ORR higher than in previous studies and without significant toxicities^23^. However, these studies only focused on the clinical outcome of treatment with the third-generation anti-CD30 CAR T-cells, and did not investigate the specific biology, in vitro antitumor activity, or in vivo efficacy mechanisms between second-generation and third-generation CARs. Here, we report the third-generation anti-CD30 CAR T-cells is safe and effective in mouse models.

## Materials and methods

### Cell lines

L428 and L540(Human Hodgkin’s lymphoma cell line) were purchased from the DSMZ, Jurkat (Human T cell leukemia), K-562 (human chronic myeloid leukemia) and Raji (human lymphoblastic Burkitt's lymphoma) cell lines were purchased from CASCB (Chinese Academy of Sciences Cell Bank). Their identity was confirmed by STR loci profiling performed by the DSMZ and CASCB. All the cell lines were cultured in RPMI 1640 medium with 10% FBS. The Lenti-X 293 T cell line was obtained from TAKARA, and its identity was confirmed by STR loci profiling performed by them. Cell lines were placed in a 37 °C and 5% CO_2_ humidified incubator and tested to exclude mycoplasma contamination.

### DNA constructs and lentiviral production

The single chain fragment variable (scFv) nucleotide and CAR sequence of CD30 was derived from our proprietary product (patent number: CN106589139B). The construction of the CAR is shown in Fig. 2A. Production of clinical-grade lentiviral vectors was performed with a four-plasmid system transfected into Lenti-X 293 T cells.

### CAR T-cell production

Autologous peripheral blood mononuclear cells (PBMC, from 80 to 100 ml of blood) were isolated from each patient by low-density centrifugation on lymphocyte separation medium (GE healthcare, USA), and T lymphocytes were separated from these using MACS human CD3 microbeads (Miltenyi Biotec GmbH, Germany) following the manufacturer’s instructions. After activation overnight with Dynabeads Human T-Activator CD3/CD28 (Gibco, USA), T cells were transduced with lentivirus at a multiplicity of infection (MOI) ranging from 3 to 5. Transduction efficiencies were monitored by flow cytometric analysis after 3 days. All the CD3+ T cells were cultured using TexMACS GMP medium with additional IL-2, HEPES and L-Glutamine.

### CAR T-cell copy number assay

The numbers of copies of CAR gene on anti-CD30 CAR-T cells was determined by TaqMan quantitative PCR assay. Genomic DNA (gDNA) were isolated using QIAamp DNA Blood Mini Kit (Qiagen) according to the manufacturer’s instructions. DNA concentration was measured by UV spectroscopy (Q5000; Quawell). Samples were diluted to a final concentration of 50 ng/uL in nuclease-free H2O. Primers targeting the CAR transgene were used:forward primer:5′-CAGGACTCGGCTTGCTGAA-3′,reverse primer:5′-AATACTGACGCTCTCGCACC-3′,probe:5′-CGGCGACTGGTGAGTACGCCAAA-3′.To normalize the DNA amounts for the qPCR assay, we used TaqManTaqMan Gene Expression assay HBB (Applied Biosystems, 4331182) in another independent qPCR reaction mix. The HBB gene has two copies per diploid cell and is used as a reference gene. Thermal cycling for all PCR experiments was performed using the following amplification conditions on the CFX Connect Real-Time PCR System(Bio-rad): Denaturation for 10 min at 95 °C,followed by 44 cycles at 95 °C for 15 s and 60 °C for 34 s.A six-point standard curve was generated via serial dilution of mCD30 plasmid DNA and β-globin plasmid DNA.Standard curves were included in every experiment and met predefined criteria (qPCR efficiency between 90 and 110%, adjusted R2 ≥ 0.99). Each data point was evaluated in triplicates with mean values used for analysis. The copy number of anti-CD30 CAR-T cells was calculated by the following formula: CAR copy number/cell = 2 × CAR gene copy number/HBB gene copy number.

### Flow cytometry

The lentivirus titer and cell transduction efficiency were monitored using a Beckman CytoFLEX FCMS. CD30 expression on cells was analyzed using the following antibodies: CD30 (APC anti-human CD30, clone BY88, Biolegend) and the isotype control (Mouse IgG1, κ; Biolegend). Cell samples were collected and incubated with antibodies at the recommended dilution at 4 °C for 30 min, then washed twice with PBS (Gbico, USA) containing 2% FBS (Gibco, USA) after staining. FACS data were analyzed with FlowJo software (TreeStar V10).

### Cytotoxicity assay

The cytotoxicity of CAR T-cells was measured by the calcein release assay. Targeted cells were labeled with 25 μM Calcein-AM (Aladdin) at 37 °C for 30 min, and then co-cultured with anti-CD30 CAR T-cells (at the indicated effector: target ratios of 25:1, 5:1, 1:1) in 96-well plates for 2.5 h. The supernatant was harvested and fluorescence intensity (FI) quantified using a microplate reader (PerkinElmer Victor X3) with an excitation wavelength of 485/20 and emission wavelength 530/25. The tumor killing efficiency was calculated as previously reported^24^.

### Xenograft mouse model

Eight-week-old NPG mice were purchased from the Beijing Vitalstar Biotechnology Co, Ltd. For the first mouse xenograft model, 1 × 10^6^ L428 cells were intravenously injected into each mouse. Tumor progression was monitored by analysis of the ratio of L428 cells in the mouse PBMC using FACS. On day 6 after L428 cell injection, the mice were divided into three groups and were intravenously injected with 1 × 10^7^ anti-CD30 CAR T-cells, untransfected T cells or PBS alone. For the second mouse xenograft model, 1 × 10^6^ L428 cells were injected subcutaneously into the unilateral axillary region of NPG mice. The tumor volume was measured twice a week by Vernier calipers. After the tumor volume reached approximately 20 mm^3^, the mice were randomly assigned to four groups, PBS alone, 1 × 10^7^ untransfected T cells, ,1 × 10^7^ anti-CD30 CAR T-cells (CD30-28z and CD30-Z8BBz) and the indicated number of CAR T-cells was infused via the caudal veins of the mice. We also collected the different organs from different stages during tumor progression and assessed the CAR copy number at each time in the CD30-Z8BBz group. On day 25, 3 or 5 mice per group were decapitated and paraffin sections of the liver, spleen and other important tissues were made for histopathology as previously reported^25^. All animal experiments were approved by the Animal Ethics Committee of Wuhan University of Science and Technology, Wuhan, China. All experiments were performed at the animal experimental center of Wuhan University of Science and Technology. All methods were carried out in accordance with relevant guidelines and regulations and reported in accordance with ARRIVE guidelines.

### Tumorigenicity

NPG mice were subcutaneously injected once with negative control cells (normal lung tissue cell, MRC-5 cell line), positive control cells (Hela cells), low dose (1 × 10^6^) or high dose (1 × 10^7^) of anti-CD30 CAR T-cells (CD30-28BBz). The animals were sacrificed on day 112 for evaluation of tumorigenic potential of the anti-CD30 CAR T-cells by H&E staining of different tissues from the different mouse models. All animal experiments were approved by the Animal Ethics Committee of Wuhan University of Science and Technology, Wuhan, China. All experiments were performed at the animal experimental center of Wuhan University of Science and Technology. All methods were carried out in accordance with relevant guidelines and regulations and reported in accordance with ARRIVE guidelines.

### Membrane proteome array specificity testing

Membrane Proteome Array (MPA) screening was conducted at Integral Molecular, Inc. The MPA is a protein library composed of 5300 distinct human membrane protein clones, each overexpressed in live cells from expression plasmids. Each clone was individually transfected in separate wells of a 384-well plate followed by a 36 h incubation^26^. Cells expressing individual MPA proteins were arrayed in duplicate in a matrix format for high-throughput screening. Before screening on the MPA, the CD30 scFv concentration for screening was determined on cells expressing positive (membrane-tethered Protein A) and negative (mock-transfected) binding controls, followed by detection by flow cytometry using a fluorescently-labeled secondary antibody. Each test antibody was added to the MPA at a predetermined concentration, and binding across the protein library was measured on an Intellicyt iQue using a fluorescently-labeled secondary antibody. Each array plate contains both positive (Fc-binding) and negative (empty vector) controls to ensure plate-by-plate reproducibility. CD30 scFv interactions with any targets identified by MPA screening were confirmed in a second flow cytometry experiment using serial dilutions of the test antibody, and the identity of the target was re-verified by sequencing.

### Elisa

Cytokine-release assays were performed by establishing a co-culture system with 2 × 10^5^ CAR T-cells and target cells at a 1:1 ratio, and the supernatant was collected after 6- and 24-h culture. hIL-6 and hIFN-γ concentrations were determined by enzyme-linked immune-absorbent assay (ELISA Kit Product Manual; Neobioscience), and the fluorescence intensity was measured using a microplate reader (PerkinElmer Victor X3). For the mouse model, blood samples were collected from mice for measurement of plasma concentrations of cytokines at different time points. The plasma concentrations of human IFN-γ, TNF-α, IL-6 and mouse IL-6 in L428-bearing mice were measured using an enzyme-linked immunosorbent assay (ELISA) according to the manufacturer’s instructions.

### Integration site analysis

Integration site analysis (ISA) was performed on anti-CD30 CAR T-cells; processing of genomic DNA to amplify integration loci used nonrestrictive linear amplification-mediated PCR (nrLAM-PCR methods)^27^. All PCRs were performed using Platinum Taq DNA Polymerase (Invitrogen) with the primer sequences listed below. Briefly, nrLAM-PCR was initiated with a preamplification of the genomic DNA by linear PCR using 5-biotinylated primers (CD30-01-LTRI) which hybridize to long terminal repeat (LTR) regions of the CD30 CAR vectors. Then the biotinylated single-stranded PCR products were isolated by purification via magnetic streptavidin beads (Streptavidin Magnetic Beads, New England BioLabs). Next, an RNA ligase (T4 RNA ligase 1, New England BioLabs) directly ligates a known single-stranded DNA (ssDNA) linker (ssLC) to the unknown part of the PCR products. The target PCR products which include the vector-genome junction are now composed of a known linker sequence, an unknown genomic flanking sequence and a known vector sequence (LTR). Finally, the fragments can be exponentially amplified by nested PCR using vector- and linker specific primers (CD30-01-LTRII/ LCI and CD30-01-LTRIII/LCII). Purified PCR fragments were prepared for library construction (VAHTS Universal DNA Library Prep Kit for Illumina V3, Vazyme). ISA reads were sequenced using the Illumina NovaSeq next-generation sequencing platform for paired-end reads. For raw data, each sequence was trimmed using cutadapt to remove sequence reads with a quality score below 30. A new reference sequence combined the LTR sequence with the human hg19 reference gene was built. Clean reads were mapped against the new reference sequence using BWA-SW, which is a Smith-Waterman alignment tool. Integration sites were detected by using lumpy software. Raw data are available in the NCBI Sequence Read Archive (SRA) database under the BioProject number PRJNA656395.

### Statistical analysis

Prism 5.0 (GraphPad Software) was used for data analysis. Two-way analysis of variance (ANVOVA) was used to determine the significance of the differences between means in all experiments. Survival curves were generated using the Kaplan-Meier method. p value <0.05 was considered statistically significant.

### Ethics approval and consent to participate

This study was approved by the Animal Ethics Committee of Wuhan University of Science and Technology of ID WKD-Zhu-1.

## Results

### Specificity of scFv for CD30

**Figure 1:**
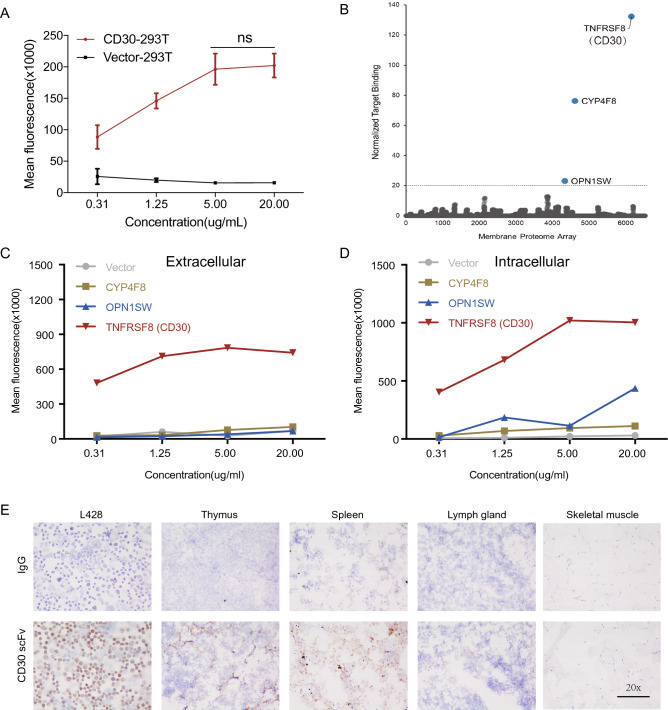
The scFv recognition of CD30 antigen. **(A)** Binding to the CD30 antigen is increased with increasing concentrations of scFv with 5 μg/ml being the optimal concentration. **(B)** Binding of scFv to 293 T cells individually expressing 5300 human membrane proteins and measured by Facs analysis using a fluorescently-labelled antibody. Three membrane proteins (TNFRSF8, also known as CD30, CYP4F8 and OPN1SW) were identified as the candidate binding proteins for scFv. **(C,D)** Cell surface scFv binding to the CD30 (TNFRSF8) molecule (left), but the other two candidate protein targets bound intracellulary. **(E)** Immunohistochemistry analysis of normal human tissue stained with scFv antibody (IgG as the negative control).

In previous studies, CD30 was found to be highly expressed by almost all Hodgkin lymphomas (HL), anaplastic large cell lymphomas (ALCL) and subsets of T cell lymphomas (TCL)^28^ which is consistent with the Cancer Cell Line Encyclopedia (CCLE) analysis (Supplemental Fig. 1, data modified from the website of https://portals.broadinstitute.org/ccle). However, limited expression by a subset of active T/B cells, indicates that CD30 would be an excellent candidate target for CAR T-cell therapy. To validate the antigen recognized by the antibody we used in this study, we performed CD30 scFv screening on the entire array to identify positive binding using the Membrane Proteome Array. This is a platform for profiling the specificity of antibodies and other ligands that target human membrane proteins which can be used to determine antibody target specificity and deconvolute orphan antibody/ligand targets. To optimize conditions for test antibody detection, HEK-293 T cells were transfected with plasmids encoding CD30 or control vector. The average mean fluorescence reflecting the binding capability increased with increasing CD30 scFv concentrations (0.31–5.0 μg/ml) and tended to plateau after reaching 5.0 μg/ml, whereas there was no signal in the control vector group (Fig. 1A). After the saturating concentration of CD30 scFv (5.0 μg/ml) had been established, further studies were undertaken to determine whether the CD30 scFv displayed cross-reactivity on other human membrane proteins, which was very important to exclude before this antibody could be used for CAR T generation. Plasmids containing cDNA clones of 5,344 membrane proteins (representing over 90% of the human membrane proteome, including CD30) were each reverse transfected into HEK-293 T cells in 384-well cell-culture plates (Supplemental Table 1). Test antibodies were added to Membrane Proteome Array matrix plates at pre-determined concentrations and detected by flow cytometry with data captured using ForeCyt Software (Intellicyt). As expected, MPA screening revealed that the scFv we selected was highly specific for its target, TNFRSF8 (CD30) (Fig. 1B). Interestingly, the off-target interactions which were detected for CD30 scFv were low-level reactivities to other membrane proteins, such as CYP4F8 and OPN1SW, which are proteins with an intracellular localization reaction but are not detected in the extracellular assay (Fig. 1C,D).

After the scFv was confirmed to specifically recognize CD30 with high sensitivity, we purified the scFv with his-tag and characterized for cross-immunoassay detection on 34 different human organs using frozen sections. The CD30 scFv strongly stained the positive control (L428) and did not react with the negative control (skeletal muscle) (Fig. 1E, left). We also observed CD30 scFv-positive signals in the spleen, lymph nodes and thymus, where immune cells accumulate (Fig. 1E, right). We observed no intracellular or extracellular signals in another 29 human tissues, except for some non-specific background staining present in the bone marrow, colon, ileum and stomach (Supplemental Fig. 1B). The overall results indicated that the scFv was a good candidation for CAR-T therapy according to its specific target molecule recognition and limited binding to normal tissue.

### Anti-CD30 CAR T-cells specifically target CD30-positive cells

**Figure 2:**
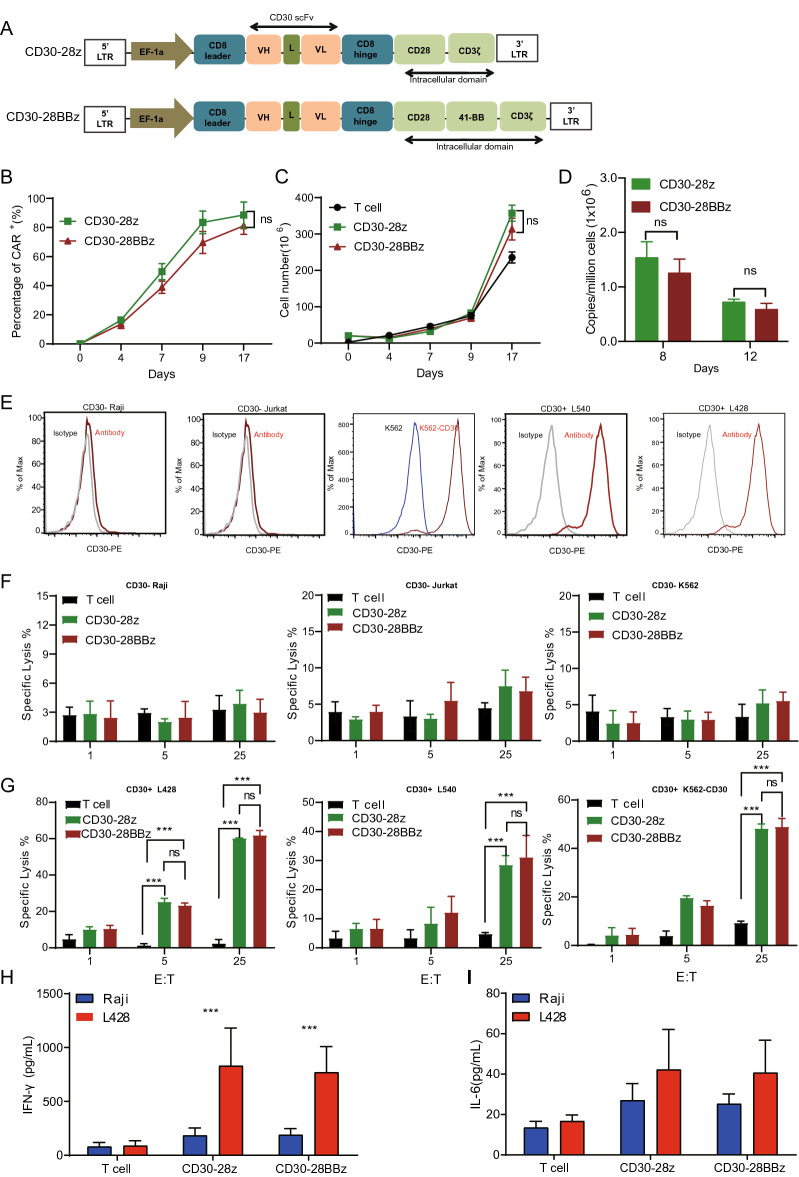
Proliferation and cytotoxicity of anti-CD30 CAR T-cells. **(A)** Schematic illustration of the anti-CD30 CAR (CD30-28z and CD30-28BBz) constructs. **(B)** Percentage of CAR+ T cells during the CAR T-cells (CD30-28z and CD30-28BBz) in vitro culture. **(C)** Total cell number at the time of transduction of anti-CD30 CAR T-cells (CD30-28z and CD30-28BBz). **(D)** The transgene copy number per one million CARS+ cells at the time of transduction of anti-CD30 CAR T-cells (CD30-28z and CD30-28BBz). **(E)** FACS was used to detect CD30 expression in negative cells (Raji, Jurkat and K562) and positive cells (L428 and L540). Gray line: isotype control; Blue line: K562; Red line CD30 antibody and K562 overexpression CD30. **(F)** The calcein release assay was used for in vitro cytotoxicity testing at 3 different effectors: target ratios on CD30 negative cell lines as indicated. **(G)** The calcein release assay was used for in vitro cytotoxicity testing at 3 different effectors: target ratios on CD30 positive cell lines and K562-CD30 as indicated. **(H–I)** Anti-CD30 CAR T-cells were co-cultured with L428 cells. After 24 h, supernatants were collected for cytokine(IFNγ and IL-6) production analysis. All experiments were performed at least three independent times, and *p ≤ 0.05; **p ≤ 0.01; ***p ≤ 0.001. In the (**B,C,D,F,G**) black bar: T cells; green bar: CD30-28z; red bar: CD30-28BBz.

For the second and third generation anti-CD30 CAR, a lentivirus vector incorporating the anti-CD30 scFv, CD8-derived hinge and transmembrane regions, only CD28 (28z) or coupled to the CD28 and 4-1BB (28BBz) co-activation domains and linked to the CD3ζ activation domain were constructed, respectively (Fig. 2A). T cells were activated with anti-CD3/CD28 monoclonal antibody beads and transduced with lentivirus encoding the 28z and 28BBz and strep tag II which was used to monitor CARs transduction efficiencies. The results showed that the number of the CAR-positive cells was low at the 3 days but increased by the 12 days, indicating that CAR positive cells have greater amplification potential than negative cells (Fig. 2B, Supplemental Fig. 2A). However, there was no significant difference in CAR expression and cell amplification between 28z and 28BBz in vitro (Fig. 2 B,C, Supplemental Fig. 2A). All of the anti-CD30 CAR T-cells expanded by an average of 200-fold by day 14 of culture (Fig. 2C). Furthermore, we performed qPCR to evaluate the transgene copy number per cell and showed stable vector integration during the anti-CD30 CAR T-cells culture. This was also not different between 28z and 28BBz CAR T cells (Fig. 2D). To assess whether our CARs transduce different intracellular signals which could affect the tonic CAR signaling, we analyzed the T cell subtype and phenotype during the primary expansion. Both of CD28z and CD28BBz CAR T cells were similar regarding T cell subsets distribution, T cell activity, effector/memory and exhaustion, suggesting that 4-1BB proximity to the cell membrane did not affect the tonic signaling phenotype in the anti-CD30 CAR T-cells in vitro cultures (Supplemental Fig. 2B–E).

Next, to compare the cytotoxicity of between 28z and 28BBz the CD30-positive cell lines L428, L540, and the CD30-negative cell line Raji, Jurkat and the K562 cell lines artificially overexpressing CD30 were used for further anti-tumor activity studies. The CD30 expression by each cell line was confirmed by FACS analysis (Fig. 2E). Calcein-AM based cytotoxicity assays demonstrated that both of the 28z and 28BBz cells lysed over 50% of the L428 cells at an E:T ratio of 25:1, whereas Raji, Jurkat and K562 cells were not killed to any significant degree cmpared to control T cells and 28z and 28BBz (almost zero killing in both cases) (Fig. 2F,G). Similar results obtained for L540 cells and CD30 overexpressing cell line K562 (Fig. 2G). We found CD28 CAR-T and 28BBz CAR-T cells displayed higher tumor lysis capacity than 4-1BBz CAR-T cells in vitro at an E:T ratio of 25:1(Supplemental Fig. 2F) and then selected CD28 CAR-T and 28BBz CAR-T cells for in vivo antitumor assays.

When co-cultured with L428 cells, but not with Raji cells, anti-CD30 CAR T-cells were found to increase IFN-γ secretion but again with no significant difference between 28z and 28BBz (Fig. 2H). In addition, these cells produced similar levels of IL-6 following co-culture with CD30-positive/negative cells (Fig. 2I). These data demonstrated that anti-CD30 CAR T-cells specifically target CD30-positive cells and that there were no significant differences between second and third generation CAR T-cells in in vitro studies.

### In vivo activity of CD30-targeted CAR T-cell therapy

To evaluate the in vivo activity of anti-CD30 CAR T-cells, we performed experiments using two NPG (NOD.Cg-prkdcscid Il2rgtmlwjl/SzJ) xenograft models created by different mouse modeling strategies. In the first model, NPG mice were injected with L428 cells via the tail vein. After engraftment of the tumor cells for 3 days, the mice were treated either with a single dose of PBS, 3 × 10^6^ control T cells or 3 × 10^6^ anti-CD30 CAR T-cells (CD30-28BBz). It was found that the CD30-28BBz significantly prolonged survival compared with vehicle or control T cells (Supplemental Fig. 3A). At 120 days after CD30-28BBz treatment, approximately 50% of the mice were still alive.

**Figure 3:**
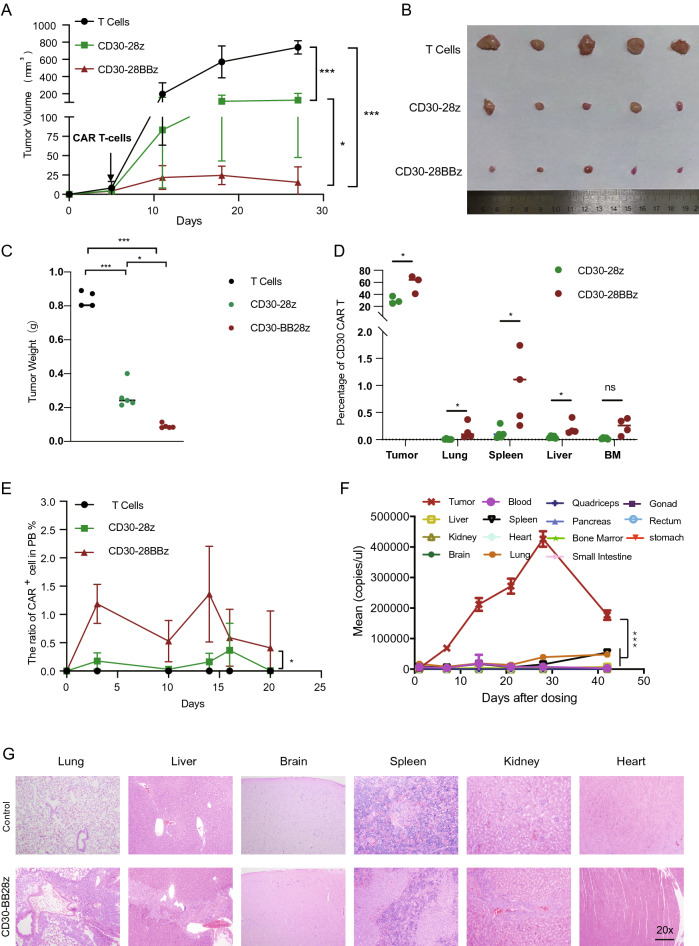
In vivo anti-tumor activity of anti-CD30 CAR T-cells. **(A)** Effect of anti-CD30 CAR T-cells (CD30-28z and CD30-28BBz) on tumor volume in L428 xenografts (n = 5). **(B,C)** Spleens from different treatment groups were weighed(B) and photographed(C) in L428 derived mice model(n = 5). **(D)** Percentage of anti-CD30 CAR positive cells in tumour and different mouse organs. **(E)** Detection of anti-CD30 CAR T-cells in the peripheral blood using flow cytometry for mouse model as the time indicated. Cells were gated on T lymphocytes identified as hCD45+ and hCD3+ cells. **(F)** Anti-CD30 CAR T-cell expansion and persistence in different organs. Three mice were sacrificed at each of several serial time points and q-PCR was performed to determine the expression of the CAR in different organs. **(G)** Images of H&E-staining of lung, liver, brain, spleen, kidney and heart tissue from mice receiving untransfected T cells or anti-CD30 CAR T-cells (CD30-28BBz). All experiments were performed at least three independent times, and *p ≤ 0.05; **p ≤ 0.01; ***p ≤ 0.001. **(A–E)** Black bar: T cells; green bar: CD30-28z; red bar: CD30-28BBz.

However, the Hodgkin lymphoma tumor cells are in the lymph nodes typically which are solid tumor of the immune system^29^. Therefore, we believe that subcutaneous inoculation is a more accurate model reflect the tumor environment than tail vein inoculation xenograft models. Then, we investigated the antitumor efficacy of third generation compared with the second generation anti-CD30 CAR T-cells in another xenograft model. L428 cells were subcutaneously implanted into the flanks of NPG mice to establish another xenotransplanted tumor model. When the mean tumor volume reached approximately 20 mm^3^, mice were injected with CD30-28z, CD30-28BBz and control T cells, respectivity. Notably, Both CD30-28z and CD30-28BBz prevented tumor growth compared to the rapid tumor growth in the control T cell animals (Fig. 3A–C) but not influenced the mice weight (Supplemental Fig. 3B). Interestingly, mice treated with CD30-28BBz displayed a greater number of the CAR T cells, relative to mice treated with CD30-28z, which also correlated directly with their antitumor activity (Fig. 3D).

To evaluate the persistence and homing capability between the CD30-28z and CD30-28BBz, qPCR was used to measure CAR copy numbers in PB on different days after treatment. The results showed that CD30-28BBz persisted was longer than CD30-28z (Fig. 3E). We also observed that the CD30-28BBz persisted more longer than CD30-28z in different organs such as the liver, spleen and others, as well as the tumor itself after mice sacrificed (Fig. 3D). Furthermore, the copy numbers of CAR indicated robust cell expansion in the tumor compared to the small number of CAR transcripts observed in the other organs on different days after treatment (Fig. 3F). The most common acute toxicity associated with CAR T cell therapy is cytokine release syndrome (CRS). The main source of the key CRS cytokine interleukin 6 (IL-6) is macrophages and monocytes^30^. We found that mIL-6 come from mouse cells or IL-6 from the CAR-T cells(hIL-6) which maintainance at a low level after CD30-28z and CD30-28BBz treatment (Supplemental Fig. 3C,D). Furthermore, CD30-28BBz produced more amount of INF-γ than CD30-28z while not significant for the TNF-α(Supplemental Fig. 3E,F). In addition, the exhaustion status of T cells was analyzed in tumor site. Compared with CD30-28z CAR T-cells, significant fewer CD30-28BBz CAR T-cells expressed PD-1 and TIM-3, whereas no significant difference for LAG-3 and CTLA-4 expression (Supplemental Fig. 3G).

Additionally, common CAR T-related toxicities, such as cytokine release syndrome (CRS), neurological toxicity, on-target/off tumor and off-target toxicities, which involved in all organs and tissues^31^. To evaluate the toxicity of CD30-28BBz, some major organs from xenograft mice were examined by H&E staining. Compared with the normal T cell treatment group, no significant difference was observed in spleen, kidney, liver, and heart from the CD30-28BBz-treated animals (Fig. 3G). Taken together, our data document effective trafficking of CD30-28BBz to the tumor and mediation of significant anti-tumor efficacy compared to CD30-28z in vivo.

### Systemic analysis of anti-CD30 CAR T-cell toxicity

**Figure 4:**
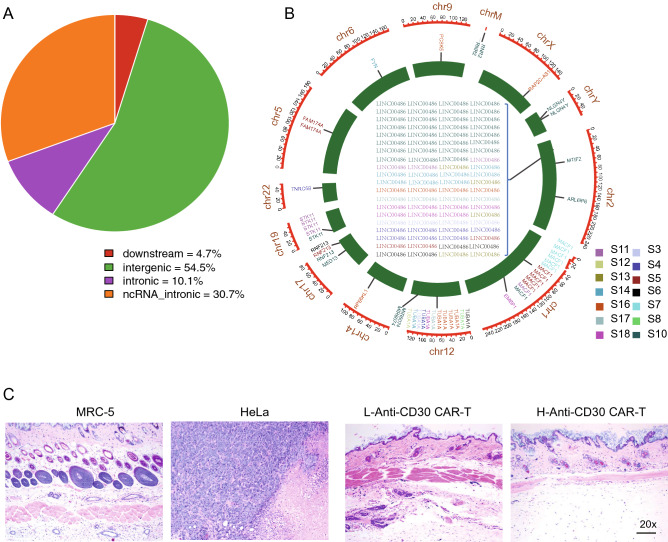
Anti-CD30 CAR-T do not have an increased risk of malignant transformation in vitro or in vivo. **(A)** The pie chart shows the overall insert integrity of the anti-CD30 CAR T(CD30-28BBz) lentiviral vector. **(B)** Circos plot visualization of the integration sites across the genome and local genomic features from the inner to outer circles. Genes that harbor these integration sites and the color of the gene names show that the integration sites probably derive from different samples. **(C)** Analysis of the tumorigenic potential of anti-CD30 CAR T cells (CD30-28BBz). MRC-5 and HeLa were used as the negative and positive control, respectively. H&E-stained images.

Available viral vectors including lentivirus and retrovirus are considered to be suitable tools for the transduction of CARs into T cells to generate CAR T-cells, but insertional mutagenesis caused by their integration is a potential risk for CAR-T therapy^32,33^. To evaluate whether the lentiviral vectors we used carried an oncogenic potential, we performed lentiviral integration site analysis of 14 anti-CD30 CAR T-cells (from patients who were infused with anti-CD30 CAR T-cells therapeutically) by nrLAM-PCR method^27^. In total, 124 integration sites (Supplemental Table 2) were identified and almost 95% of these were sites enriched at the intergenic and intronic regions, whereas no integration sites were located in the exonic, 3’UTR and upstream regions which are essential for gene expression (Fig. 4A). Additionally, to examine the features of these integration sites, we visualized each individual integration site on a circos plot (Fig. 4B). The results showed that the lentiviral integration pattern favors sites that are enriched in LINC00486, MACF1 and TUBA1A amongst others, which are located far away from the oncogenes in the T cell malignacies that were previously identified^34,35^. When CAR T was evaluated by intravenous injection in vivo, no infinite expansion of CAR T was observed (Fig. 3D–F). Next, to further confirm that the anti-CD30 CAR T-cells did not have the potential to cause tumorigenicity, two concentrations of CAR T-cells were injected subcutaneously into NPG mice (n = 6 for each group). The histological results (H&E staining) on the subcutaneous tissue showed no significant changes in 112 days after CAR T-cells injection, whereas HeLa cells showed significant tumorigenicity (positive control group, 63 days) (Fig. 4C). Overall, our data further support the conclusion that anti-CD30 CAR T-cells have no side effects or potential risks of tumorigenesis.

## Discussion

Both second and third generation anti-CD30 CAR T-cells therapy had been demonstrated to mediate anti-tumor activity in phase I/II studies in refractory or relapsed HL and ALCL^17–19^. Most of these studies focused on clinical efficacy, leading to limited knowledge about the difference of CAR activity between second and third generation CAR T cells based on CD30 antigen targeting. In the present study, we described that 4-1BB-CD28-containing CD30-CARs conferred superior anti-tumor activity and tumor homing over second-generation CD28-containing CARs.

CD30 has been proved as an excellent target for immunotherapy since it is highly expressed on tumor cells from HL and ALCL^36^. However, our data showed that the anti-CD30 scFv can recongnize a small fraction of lymphoid cells in thymus, spleen and lymph node, which indicated that anti-CD30 CAR T-cells therapy may impair host cell-mediated immunity after infusion. None of the patients treated with anti-CD30 CAR T-cells experienced significant loss of lymphoid cells, which was consistent with the previous study reporting overall acceptable safety of anti-CD30 CAR T-cells therapy^23^. In addition, some positive signals for anti-CD30 scFv were also observed in the cells localized on the surface of human organs, such as adrenal medulla cells and pancreatic epithelial cells, which was consistent with the previous study that found low expression of CD30 in the pancreas resulted in serious adverse events (pancreatitis) by Brentuximab vedotin treatment^37,38^. Therefore, the off-target risk in pancreas and adrenal medulla should be carefully considered after anti-CD30 CAR T-cell infusion in future clinical studies.

CAR molecule consists of a scFv, a spacer domain, a transmenbrane domain and cytoplasmic domain, which all indispensably contribute to the maximal anti-tumor activity of CAR T-cells. Based on the number of intracellular signaling domain of costimulatory molecules, CARs can be grouped into first- to third- generation^39^. Both second generation CAR containing CD28 or 4-BB and third generation CAR containing CD28 and 4-1BB have been utilized to target various antigens such as CD19^20^, BCMA^40^, LeY^41^, PSMA^42^, GD2^43^ and mesothelin^44^. However, the therapeutic difference among CARs was not analyzed side by side. Herein, two CARs (CD30-28z and CD30-28BBz) targeting human CD30 were employed to compare their therapeutic efficacy. The results showed that CD30-28BBz CAR exhibited higher anti-tumor activity, better tumor homing and longer persistence than the second-generation CAR with CD28 only (Fig. 3). Consistently, CARs with two costimulatory domains (CD28-4-BB) targeting PSMA and mesothelin showed superior tumor eradication and increased persistence in solid tumor models compared with single domain-based CARs^45,46^. A similar phenomenon was also described by Sonia Guedan et al.^45^, who discovered that combining ICOS and 4-1BB conferred CAR increased persistence and superior antitumor effect in vivo compared with 4-1BB-based CAR^45^. Besides preclinical studies, a clinical trial in 16 patients with r/r non-Hodgkin’s lymphoma showed that the third-generation anti-CD19 CAR T-cells (CD28 and 41-BB) exhibited stonger anti-tumor activity than the second-generation CAR T-cells (CD28 only) with mild cytokine release syndrome (CRS) in 6 patients^20^.

In contrast, there are several publications showed minimal enhancement of antitumor antivity or clinical benefit for third generation CARs compared with second generation CARs in the context of anti-CD19 CAR T-cells therapy in B cell malignancies^47^. Moreover, one group even reported that the second generation CAR with one CD28 co-stimulation domain was more effective than the third generation CAR containing both CD28 and 4-1BB domains^48^. Taken together, our studies and others strongly suggested that the CAR molecule should be empirically designed for each individual target/antigen for optimal CAR T cell persistence and activity to treat cancers.

Our functional studies in vitro did not reveal any remarkable differences between the third-generation CARs and second-generation CARs in terms of cell amplification, cytotoxicity and immunophenotype (Fig. 2), which was consistent with the previous study that found both second- and third-generation CARs showed similar phenotype and antitumor activity in vitro^45^. However, compared with second generation CAR, third generation CAR increased cytokine secretion (IFNγ), maintained a low exhausted phenotype and enhanced CAR T-cell tumor infiltration in mouse model. These results suggested that 4-1BB signaling is critical for CAR T cells persistence and survival in vivo. The detailed molecular mechanism is currently unclear, but several publications implies that the phosphorylation of intracellular signaling domains activates downstream targets such as NF-κB and TRAFs, which may contribute to CAR T-cell persistence and antitumor activity^49,50^.

In summary, we developed a detailed and systematic strategy to evaluate the feasibility, safety, and efficacy of anti-CD30 CAR T-cells from in vitro cells to in vivo mouse models. More important, we found that the third generation anti-CD30 CAR T-cell is a promising therapeutic approach to cure CD30 positive malignancies.

## Supplementary Information

Supplementary Figure 1.

Supplementary Figure 2.

Supplementary Figure 3.

Supplementary Legends.

Supplementary Table 1.

Supplementary Table 2.

## Data Availability

The datasets used and/or analyzed during the current study are available from the corresponding author on reasonable request.The raw data of Integration site analysis (ISA) are available in the NCBI Sequence Read Archive (SRA) database under the BioProject number PRJNA656395 and the data also deposited in the China National Center for Bioinformation/Beijing Institute of Genomics, Chinese Academy of Sciences, under accession number HRA002216 that are publicly accessible at https://bigd.big.ac.cn/gsa.
