# Supplementary figures and images for: The third-generation anti-CD30 CAR T-cells specifically homing to the tumor and mediating powerful antitumor activity
Source: Sci Rep. 2022 Jun 21;12:10488. doi: 10.1038/s41598-022-14523-0 (PMC9213494; doi:10.1038/s41598-022-14523-0)

Figure S1

A

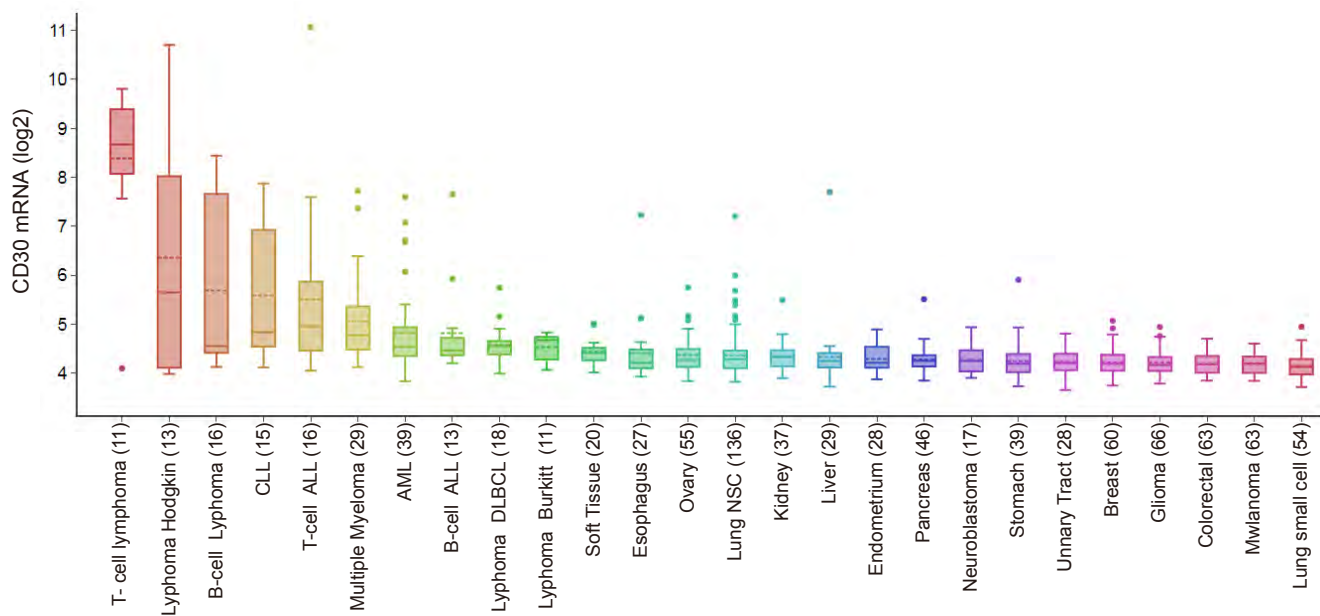

B

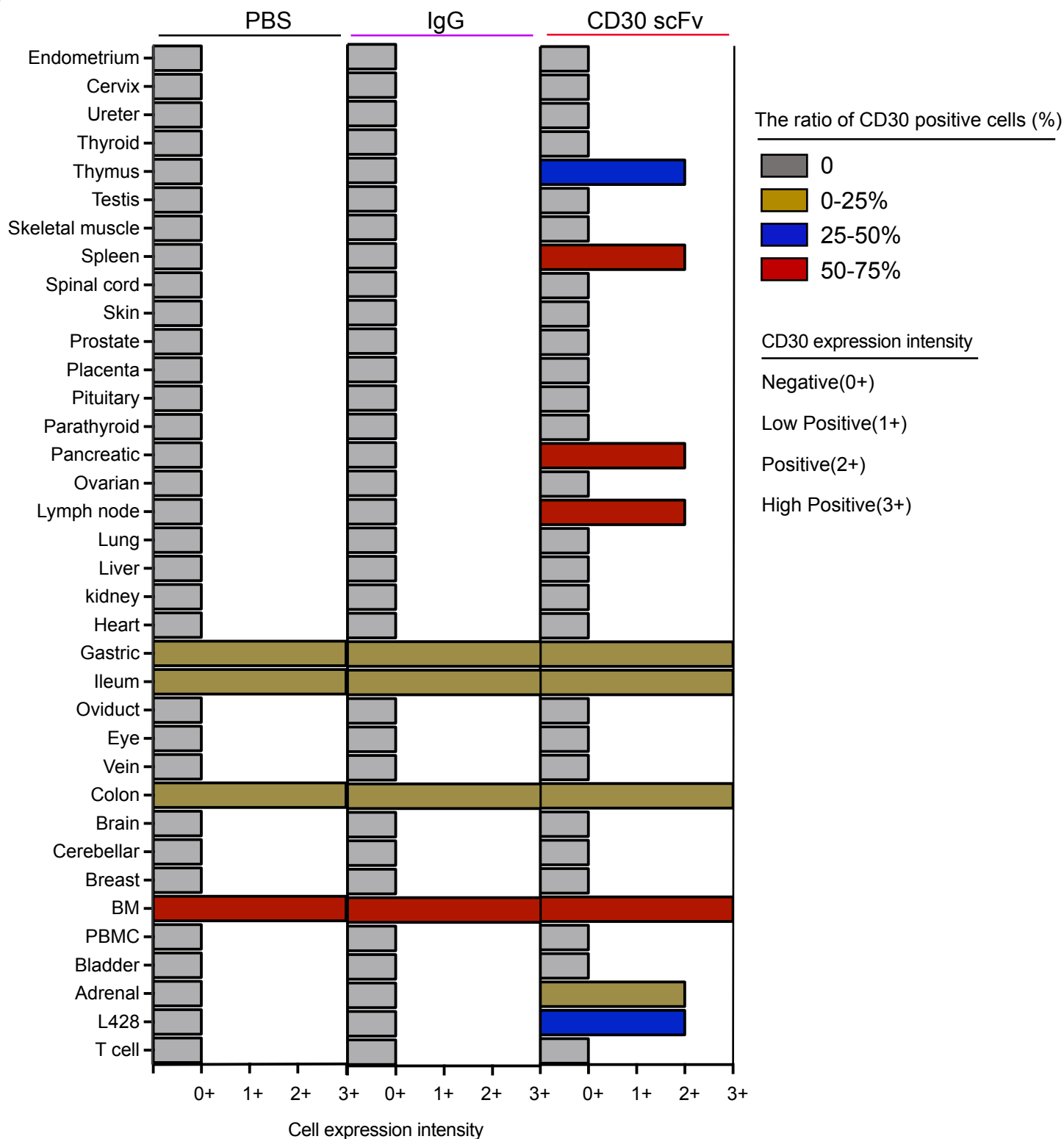

Supplement: Supplementary file 1 — Supplementary Figure 1. [file 41598_2022_14523_MOESM1_ESM.pdf]

**A**

0 days

3 days

6 days

12 days

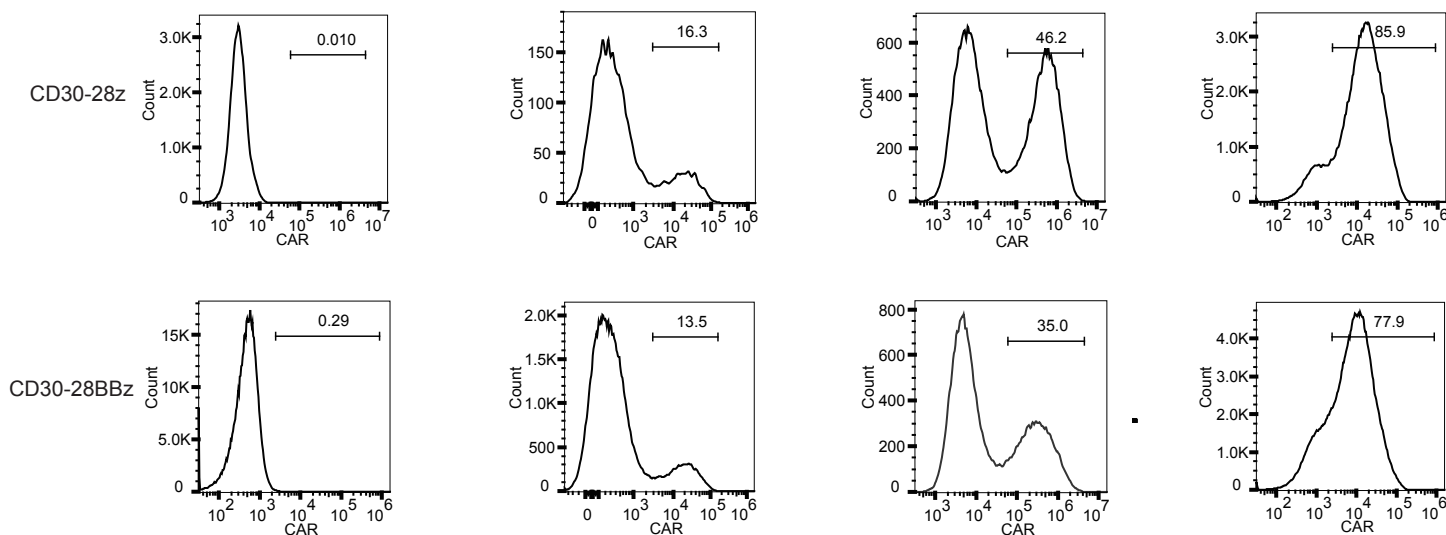**B****C****D****E**

CD30-28z

CD30-28BBz

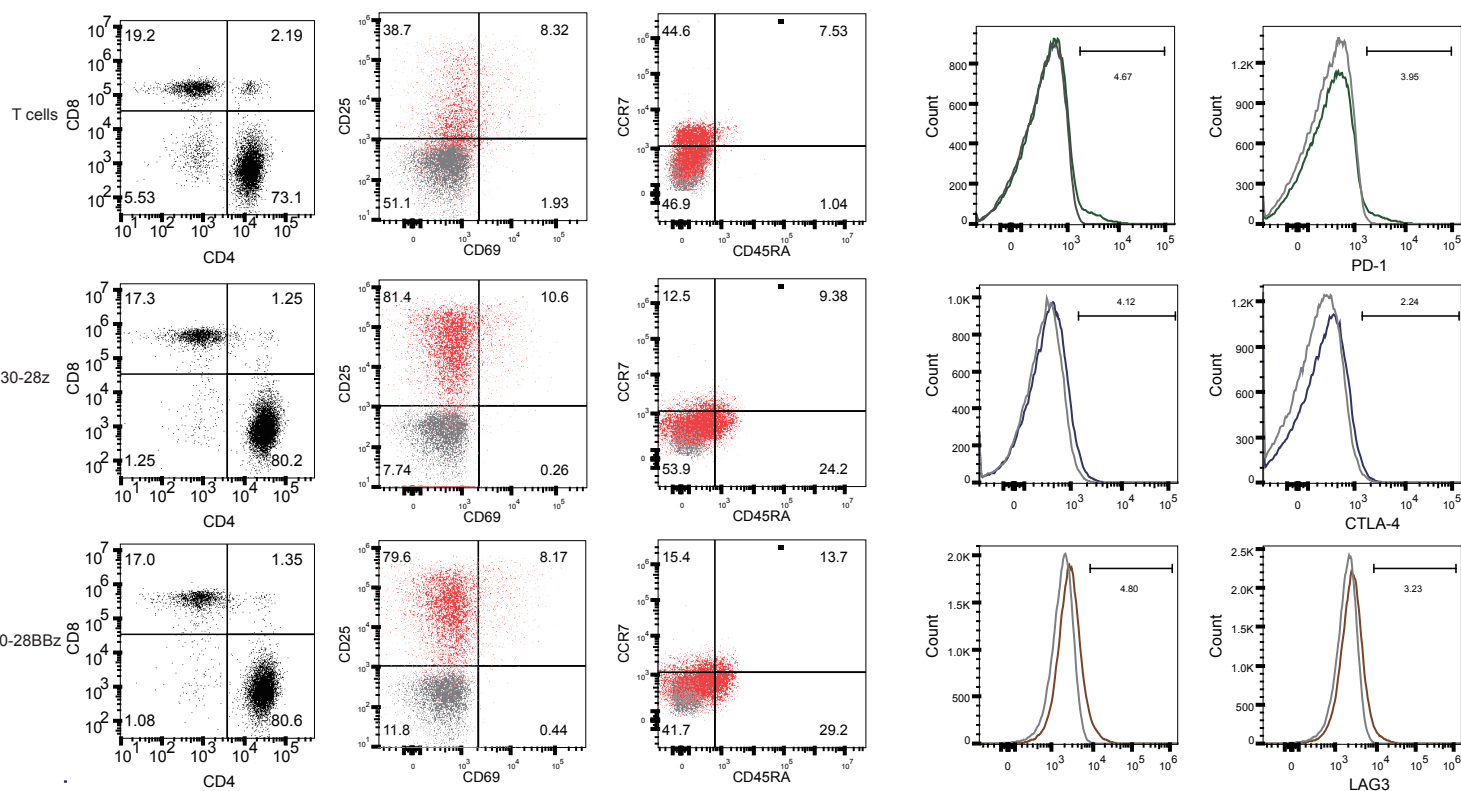**F**

K562-CD30

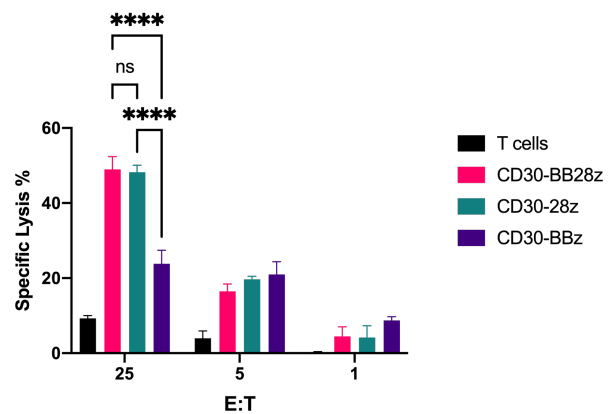

Supplement: Supplementary file 2 — Supplementary Figure 2. [file 41598_2022_14523_MOESM2_ESM.pdf]

A

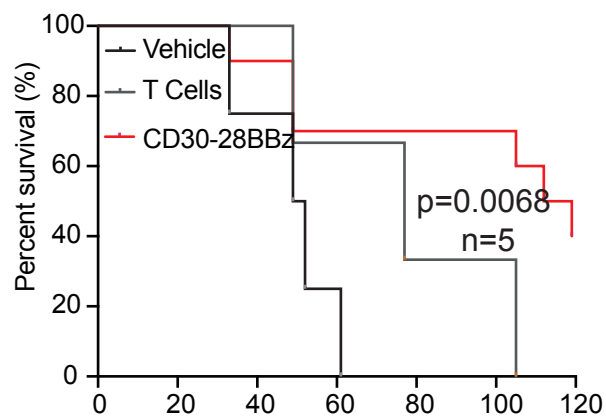

B

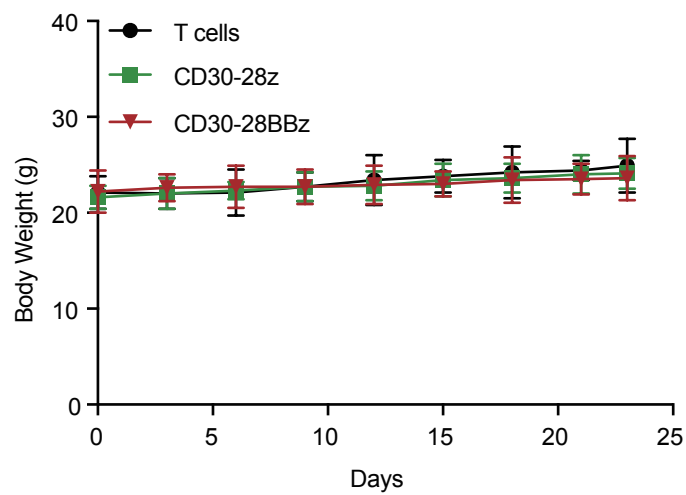

C

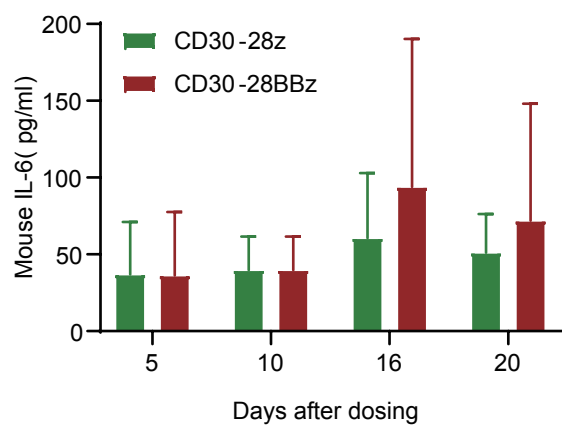

D

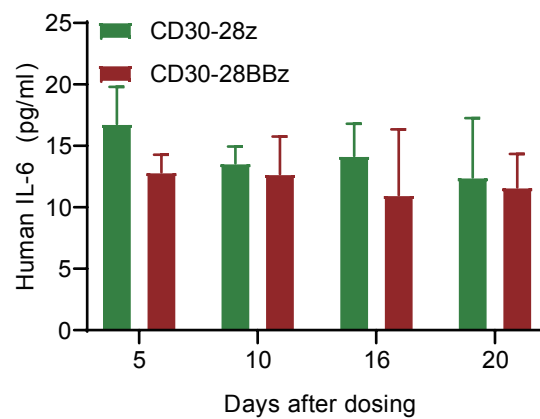

E

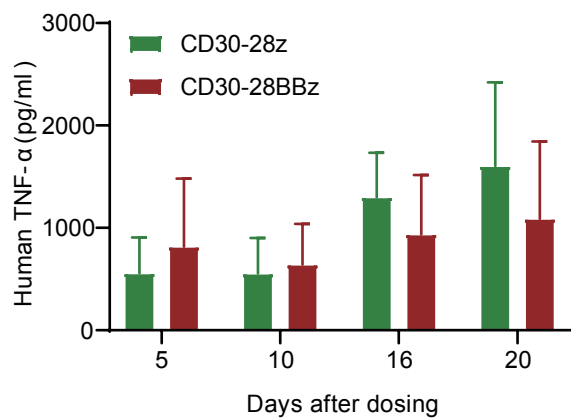

F

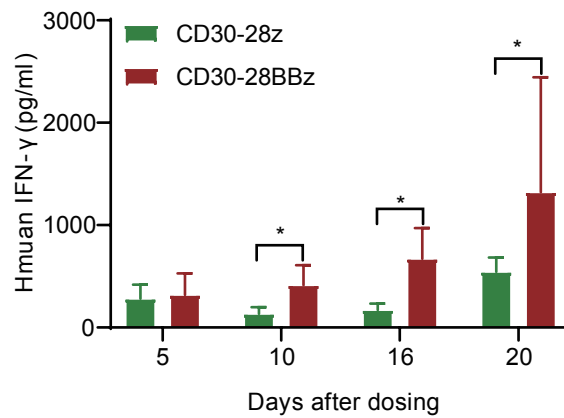

G

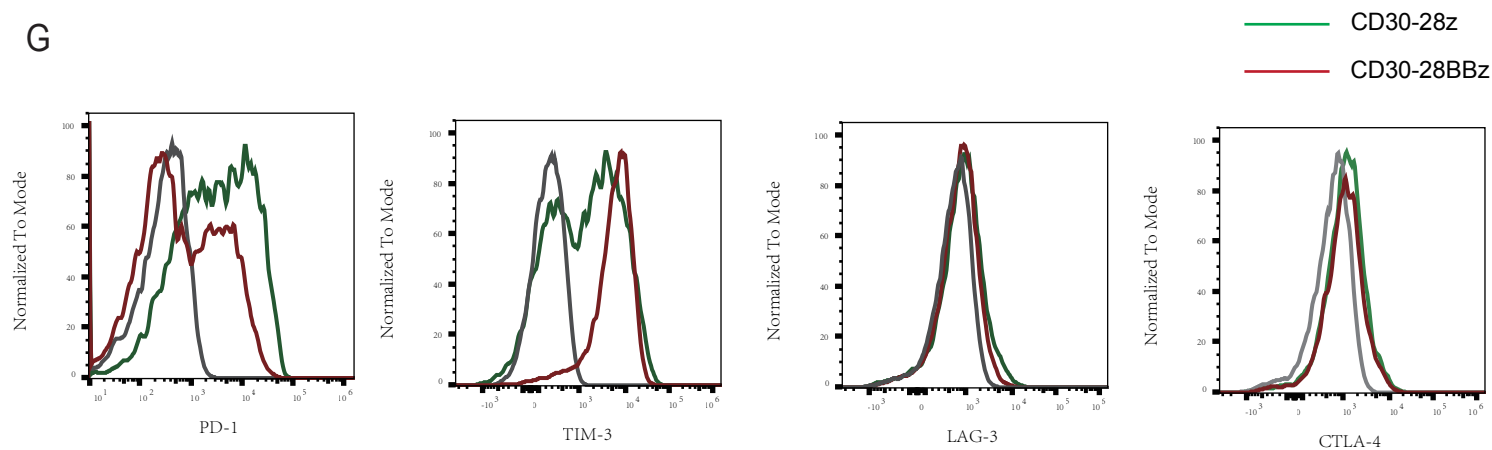

Supplement: Supplementary file 3 — Supplementary Figure 3. [file 41598_2022_14523_MOESM3_ESM.pdf]
