# Supplementary material for: The third-generation anti-CD30 CAR T-cells specifically homing to the tumor and mediating powerful antitumor activity
Source: Sci Rep. 2022 Jun 21;12:10488. doi: 10.1038/s41598-022-14523-0 (PMC9213494; doi:10.1038/s41598-022-14523-0)
Supplement: Supplementary file 4 — Supplementary Legends. [file 41598_2022_14523_MOESM4_ESM.docx]

**Supplemental Figure 1.** **(A)** CD30 expression was analyzed on 1036 human cancer cell lines from the CCLE database. **(B)** Immunohistochemistry analysis of normal human tissue stained with anti-CD30 scFv antibody (PBS, IgG as the negative control). Different colours show showed the ratio of CD30 positive cells in the organs and the number represents the intensity of CD30 expression.

**Supplemental Figure 2.** **(A)** Surface expression of the CAR proteins on different generation CAR T-cells during ex vivo expansion. **(B)** T cell subsets in different generation CAR T-cells 12 days after stimulation with anti-CD3/CD28 beads. **(C-E)** Facs analysis showing the expression of activation, differentiation and exhaustion markers in different generation CAR T-cells 12 days after stimulation with anti-CD3/CD28 beads. **(F)** The calcein release assay was used for in vitro cytotoxicity testing at 3 different effectors: target ratios on K562-CD30 cell line as indicated.

**Supplemental Figure 3. (A)** NPG mice were injected with 1x10^6^ L428 cells. Kaplan Meier survival curves of tumor-bearing mice treated with PBS, untransfected T cells or anti-CD30 CAR T-cells (CD30-28BBz) with n=5-10 mice per group. **(B)** Effect of anti-CD30 CAR-T (CD30-28z and CD30-28BBz) on the weights of mice with L428 xenografts (n=5). **(C-F)** Peripheral blood was obtained for the cytokine(mIL-6,hIL-6,hTNFα and hIFNγ) production analysis at serial time points.（**G**）Facs analysis showing the expression of exhaustion markers in different generation CAR T-cells from the tumor site after from mice receiving different treatments .

**Supplemental Table 1.**The CD30 scFv displayed cross-reactivity human membrane proteins identified by membrane proteome array.

**Supplemental Table 2.**The lentiviral integration sites of the anti-CD30 CAR T.
